# Adsorption of Cd^2+^ by an ion-imprinted thiol-functionalized polymer in competition with heavy metal ions and organic acids

**DOI:** 10.1039/c7ra11811b

**Published:** 2018-02-28

**Authors:** Qiaoping Kong, Binbin Xie, Sergei Preis, Yun Hu, Haizhen Wu, Chaohai Wei

**Affiliations:** School of Biology and Biological Engineering, South China University of Technology Guangzhou 510006 PR China hzhwu2@scut.edu.cn; School of Environment and Energy, South China University of Technology Guangzhou 510006 PR China cechwei@scut.edu.cn; Department of Materials and Environmental Technology, Tallinn University of Technology Tallinn 19086 Estonia; The Key Lab of Pollution Control and Ecosystem Restoration in Industry Clusters, Ministry of Education, South China University of Technology Guangzhou 510006 PR China

## Abstract

The simultaneous presence of heavy metals and organic acids in nature and wastewaters and their competition for adsorption sites determine the migration, transformation and fate of pollutants in the environment. A Cd^2+^-ion-imprinted polymer (Cd^2+^-IIP) with a thiol-functional group was hydrothermally synthesized by a surface imprinting technique combined with ultrasonic heating for selective adsorption of Cd^2+^ from wastewaters. The adsorbent was characterized by SEM, EDS, XPS, BET and FT-IR measurements. The experimental results concerning Cd^2+^ adsorption from single-, binary-, ternary- and quaternary-metal aqueous solutions containing Cu^2+^, Ni^2+^ and Zn^2+^ revealed high selectivity. In binary-metal solutions, relative selectivity coefficients for Cd^2+^ in respect to Cd^2+^/Cu^2+^, Cd^2+^/Ni^2+^, and Cd^2+^/Zn^2+^ were as high as 3.74, 5.73 and 4.15, respectively. In multi-metal solutions, competing heavy metal ions had little effect on the adsorption of Cd^2+^ attributed to the high selectivity of Cd^2+^-IIP towards Cd^2+^ determined by its coordination geometry. The effect of low-molecular weight organic acids on the Cd^2+^ adsorption was also studied and the results showed that the presence of tartaric, citric and oxalic acids as admixtures in Cd^2+^ aqueous solutions noticeably reduced the cation adsorption in a wide range of concentrations with the minor exception of low contents of citric and tartaric acids slightly improving adsorption.

## Abstract 2

A Cd^2+^-imprinted thiol-functionalized polymer in competition with heavy metal ions and low molecular weight organic acids was investigated.

## Graphical abstract

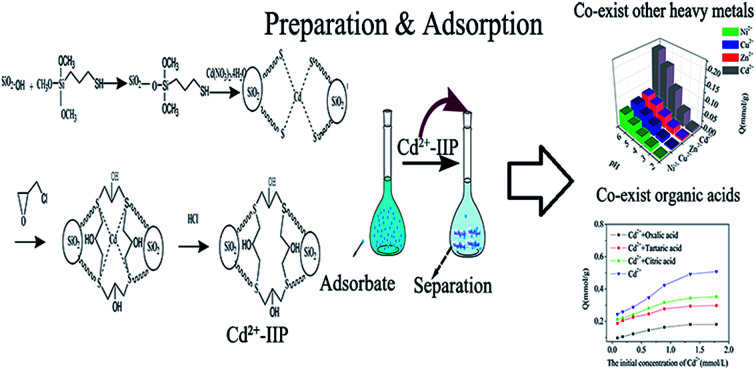

## Introduction

Cadmium, known as one of the most toxic heavy metals, brings damage to living organisms and humans even at low concentrations,^1–3^ resulting in kidney damage, impairment of enzymes, disruption of calcium metabolism, and changes in cell membrane permeability.^4^ The upper reaches of the Beijiang River in Guangdong Province have been experiencing the problem of cadmium pollution for nearly half a century due to the exploitation of mines, threatening the water supply in downstream cities. In polluted natural waters, cadmium almost never occurs alone but is accompanied with other heavy metals (Cu^2+^, Zn^2+^, Ni^2+^, Pb^2+^, Cr^3+^/Cr(vi), Mn^2+^, Fe^2+^/Fe^3+^) and organic substances (grease, oil and organic acids), competing with adsorption of the target cation.^5^ Hence, the efficient elimination and separation of cadmium from its composite wastewater is urgently required. The objective of this study is to explore a suitable adsorbent to remove cadmium form its composite wastewater. Attention towards adsorption is paid due to its simplicity, cost efficiency and the removal performance of cadmium at low concentrations,^3,6–9^ although other technologies including electrochemical,^10^ membrane filtration,^11^ ion-exchange,^12^ and selective precipitation^13^ are also used for the removal of cadmium from wastewater.

In practical applications, if adsorbents have an ability to adsorb the target pollutants from complex pollutant mixtures selectively will be better. Ion imprinting technology (IIP) is used to manufacture polymeric adsorbents with improved binding selectivity towards heavy metal ions.^14^ The selectivity of these adsorbents is achieved by the choice of specific ligands providing coordination geometry and coordination numbers suitable for the adsorbed ions, their charges and sizes.^15^ Surface ion imprinted technique is one of the most promising synthetic methods of IIP adsorbents, having advantages in simplicity, convenient preparation, and high selectivity.^16^ Recently, a few IIP adsorbents have been produced combining the surface imprinting technique with the sol–gel process, immobilizing the functional group on the surface of adsorbent material for selective removal of heavy metals from aqueous solutions.^17–19^ For example, Li *et al.*^20^ synthesized a high-selectivity 3-thiocyanatopropyltriethoxysilane (TCPTS)-based Cd^2+^-ion imprinted material IIP-TCPTS/SiO_2_ attached to the surface of silica gel particles. IIP-TCPTS/SiO_2_ showed a higher adsorption capacity and selectivity for Cd^2+^ ion than Cd^2+^ non-imprinted polymer. Singh *et al.*^21^ used Cd^2+^-ion imprinted phenol-formaldehyde-Cd^2+^-2-(*p*-sulphophenylazo)-1,8-dihydroxynaphthalene-3,6-disulphonate (PF–Cd^2+^–SPANDS) for selective adsorption of Cd^2+^ from aqueous solutions: the relative selectivities for Cd^2+^ in the presence of competing heavy metal cations comprised the row of descending order Zn^2+^ > Hg^2+^ > Cu^2+^.

In recent years, consideration attention has been devoted to study the coadsorption behavior of heavy metal ions and organic pollutants.^22^ Low molecule weight organic acids, a typical representative of organic contaminants, mainly come from the decomposition of organisms and organic matter, the response and secretion of plant roots, the metabolic synthesis of microorganisms and the emission from human activities.^23,24^ Oxalic acid, citric acid and tartaric acid are the most common three low molecule weight organic acids and they can react with heavy metals by complexation, ion exchange and adsorption, thus influencing the adsorption behavior of adsorbent to heavy metal ions.^25^ The effect of organic acids on the adsorption of heavy metals at the non-ionic imprinted adsorbents was extensively studied,^26–28^ although the ion-imprinted materials received less attention. To our best knowledge, the publications on Cd^2+^ ion-imprinted thiol-functional silica-based polymer for selective removal Cd^2+^ in consideration of both co-exist heavy metal ions (Cu^2+^, Ni^2+^ and Zn^2+^) and organic acids (oxalic acid, citric acid and tartaric acid) still remain rare. The fully understanding the interactions between ion-imprinted adsorption material and co-exist heavy metals/low molecule weight organic acids will be good for the evaluation of behavior and effects of ion-imprinted adsorbent in its practical application.

The present study considers a synthesized thiol-functional Cd^2+^-imprinted silica-based polymer Cd^2+^-IIP for Cd^2+^ adsorption competing Cu^2+^, Ni^2+^ and Zn^2+^ cations together with organic acids – oxalic, citric and tartaric. The gradient separation and removal of target Cd^2+^-ion were studied determining the factors of synergistic and inhibitory effects of the above-mentioned admixtures. An ultrasonic-assisted hydrothermal method combined with surface imprinting technique was used to prepare the Cd^2+^-IIP adsorbent. Characterization of the adsorption performance and selectivity were studied in detail.

## Materials and methods

### Reagents

Silica gel (80–100 mesh) was obtained from Qingdao Ocean Chemical Co., China. 3-Mercaptopropyltrimethoxysilane (MPTS) was provided by Shanghai Macklin Biochemical Co., Ltd., China. All the other reagents were of analytical grade and purchased from ANPEL Laboratory Technologies Inc., China.

### Preparation of Cd^2+^-IIP adsorbent

**Fig. 1:**
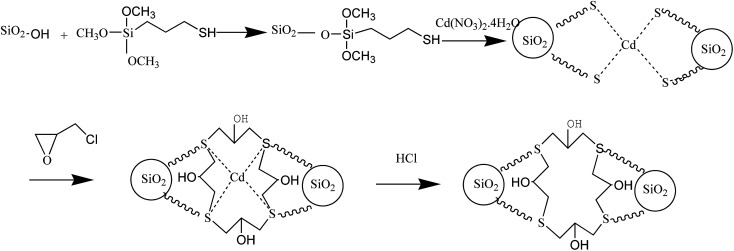
Outline of Cd^2+^-IIP adsorbent synthesis.

Silica gel in amount of 8.00 g was mixed with 60 mL of 33% methanesulfonic acid and refluxed under stirring for 8 h in order to activate the silica gel surface. The solid product was recovered by filtration, washed with distilled water to the neutral reaction, and dried under vacuum at 70 °C for 12 h. The Cd-template solution was prepared as follows: 3.08 g of Cd(NO_3_)_2_·4H_2_O were dissolved in 60 mL of methanol under stirring and heating at 40 °C for 20 min, and the solution was mixed with 4 mL of MPTS reacting for 1 h under ultrasonic heating at 60 °C. After that, 6.00 g of activated silica gel was added to the solution, sealed in a 100 mL Teflon-lined stainless steel autoclave and maintained at 120 °C for 24 h. Subsequently, 4 mL of epichlorohydrin were added to the mixture, which was then heated at 60 °C for 2 h. The product was filtered, washed with ethanol, HCl and distilled water to neutral pH, and dried at 60 °C for 24 h in vacuum. The final product of Cd^2+^-IIP was stored in the desiccator. The chemical reaction outline of preparation processes is shown in Fig. 1.

### Analytical instruments

The surface morphology of the imprinted adsorbents was examined by scanning electron microscopy (SEM, MERLIN, Carl Zeiss AG, Germany) at the desired magnification, and the equipped energy dispersal X-ray spectroscopy (EDS). Fourier transform infrared (FT-IR) spectroscopy within 4000–400 cm^−1^ wavelength using KBr pellets at 2 cm^−1^ resolution was carried out using a Nexus Por Euro FT-IR spectrometer (Thermo Nicolet, USA). Surface areas were was carried out using NOVA3200e Brunauer–Emmett–Teller (BET) surface area analyzer (Micromeritics, USA). Surface composition of the adsorbent samples was analyzed using X-ray photoelectron spectroscopy (XPS, Kratos Axis Ulra DLD, UK). The concentrations of Cd^2+^, Cu^2+^, Ni^2+^ and Zn^2+^ in aqueous solutions were measured using the AA-6300C flame atomic absorption spectrometer (FAAS, Shimadzu, Japan). Concentrations of organic acids were determined using TOC-VCPN total organic carbon analyzer (TOC, Shimadzu, Japan).

### Adsorption experiments

The adsorption experiments were carried out in the batch mode. Certain amount of Cd^2+^-IIP adsorbent was placed to a 100 mL beaker containing aqueous solutions of Cd^2+^, Cu^2+^, Ni^2+^ and Zn^2+^ ions in various combinations. After adsorption for 4 h at 25 °C, the solutions were filtered through the 0.45 μm polypropylene injection filters and the filtrate was analyzed using FAAS. The initial molar concentration ratios of heavy metal ions were set at unity for all the binary-, ternary- and quaternary-metal solutions. The solutions were adjusted to the desired pH by adding sodium hydroxide or nitric acid solutions.

To determine the adsorption capacity of Cd^2+^-IIP adsorbent in respect to organic acids, experiments were conducted with various, from 1 to 100 mg L^−1^, concentrations of the adsorbates, organic acids. The adsorption mixtures were equilibrated at pH 7.00 for 4 h. The data obtained in the experiments were used for follow-up discussion. In experiments targeting the impact of organic acids on Cd^2+^ adsorption, the adsorption of Cd^2+^ was studied at its initial concentration of 10 mg L^−1^, having organic acids dissolved in concentrations of 1–100 mg L^−1^.

### Calculations

The adsorption capacity of Cd^2+^-IIP adsorbent was calculated by eqn (1):

1 *Q* = (*C*_0_ − *C*_e_)*V*/*W*

where *Q* represents the adsorption capacity, mmol g^−1^ or mg g^−1^; *C*_0_ and *C*_e_ are the initial and equilibrium concentrations of adsorbates, mmol L^−1^ or mg L^−1^, respectively; *V* is the volume of the solution sample, L; *W* is the mass of used adsorbent, g.

The linearized forms of Langmuir^29^ and Freundlich^30^ isotherms are expressed by eqn (2) and (3), respectively:

2 *C*_e_/*Q*_e_ = *C*_e_/*Q*_max_ + 1/(*Q*_max_*K*_L_)

3 log *Q*_e_ = (1/*n*)log *C*_e_ + ln *K*_F_

where *Q*_e_ is the adsorption capacity at equilibrium, mmol g^−1^, *Q*_max_ is the maximum amount of adsorption, mmol g^−1^, *K*_L_ is the adsorption equilibrium constant, L mg^−1^. *K*_F_ is the constant representing the adsorption capacity and *n* is the constant depicting the adsorption intensity.

The distribution and selectivity coefficients of Cd^2+^ with respect to Cu^2+^, Ni^2+^ and Zn^2+^ can be obtained from the equilibrium binding data according to eqn (4) and (5):^2^

4 *K*_d_ = *Q*_e_/*C*_e_

5 *k* = *K*_d_(Cd^2+^)/*K*_d_(X^2+^)

where *K*_d_ represents the distribution coefficient; *k* is the selectivity coefficient, and X^2+^ represents competing ions of Cu^2+^, Ni^2+^ and Zn^2+^.

## Results and discussion

### Characterization of Cd^2+^-IIP adsorbent

#### SEM study

**Fig. 2:**
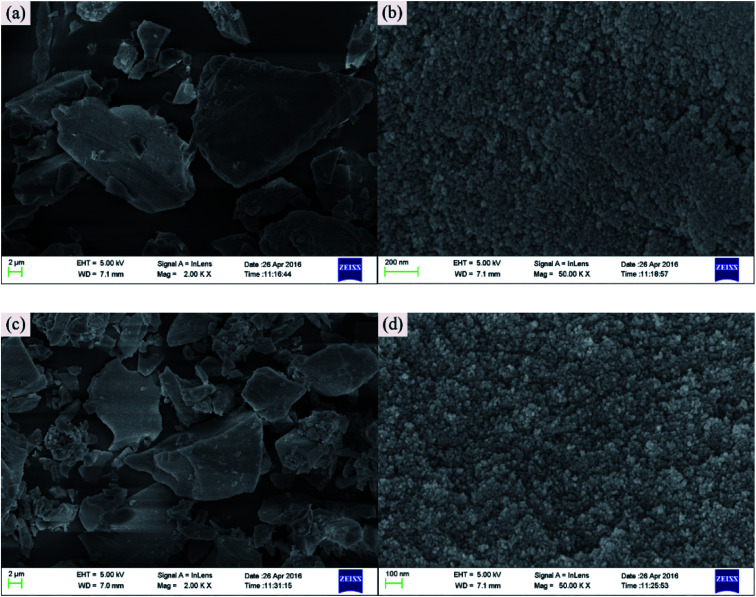
SEM photographs of activated silica gel (a, b) and Cd^2+^-IIP adsorbent (c, d).

As displayed in Fig. 2(a and b), the surface of activated silica gel was smooth, while separate aggregates were visible on the surface of Cd^2+^-IIP, Fig. 2(c and d), which left the three-dimensional network structure of silica gel unchanged as a result of surface imprinting. Compared with silica gel, the surface of Cd^2+^-IIP was fluffier and rougher. Obviously, irregular particles are seen in Cd^2+^-IIP, which might be the functional monomers that could provide sufficient recognition sites for chelating heavy metal ions. Besides, holes also appeared in Cd^2+^-IIP, the structure and size of which would determine the ion radius and type of the targeted heavy metal ions.

#### EDS study

**Fig. 3:**
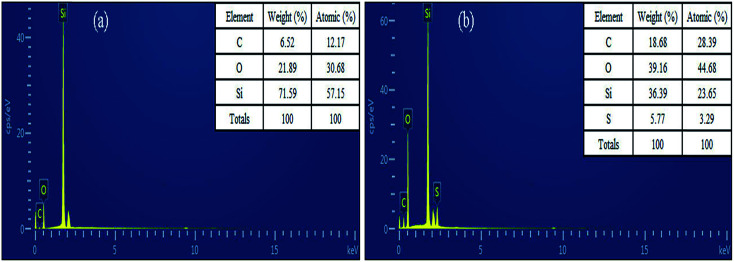
EDS analyses of activated silica gel (a) and Cd^2+^-IIP adsorbent (b).

As shown in Fig. 3, the main elements in both activated silica gel and the Cd^2+^-IIP adsorbent were silica, oxygen and carbon, to which sulfur was added as a result of thiol imprinting. The sum contents of oxygen and carbon in Cd^2+^-IIP increased from 28.41 to 57.84%, respectively, the share of silica thus expectedly decreased. Since sulfur-containing functional groups had the ability to chelate heavy metal ions, so the increment in sulfur content would improve the adsorption capacity of Cd^2+^-IIP.

#### FT-IR study

**Fig. 4:**
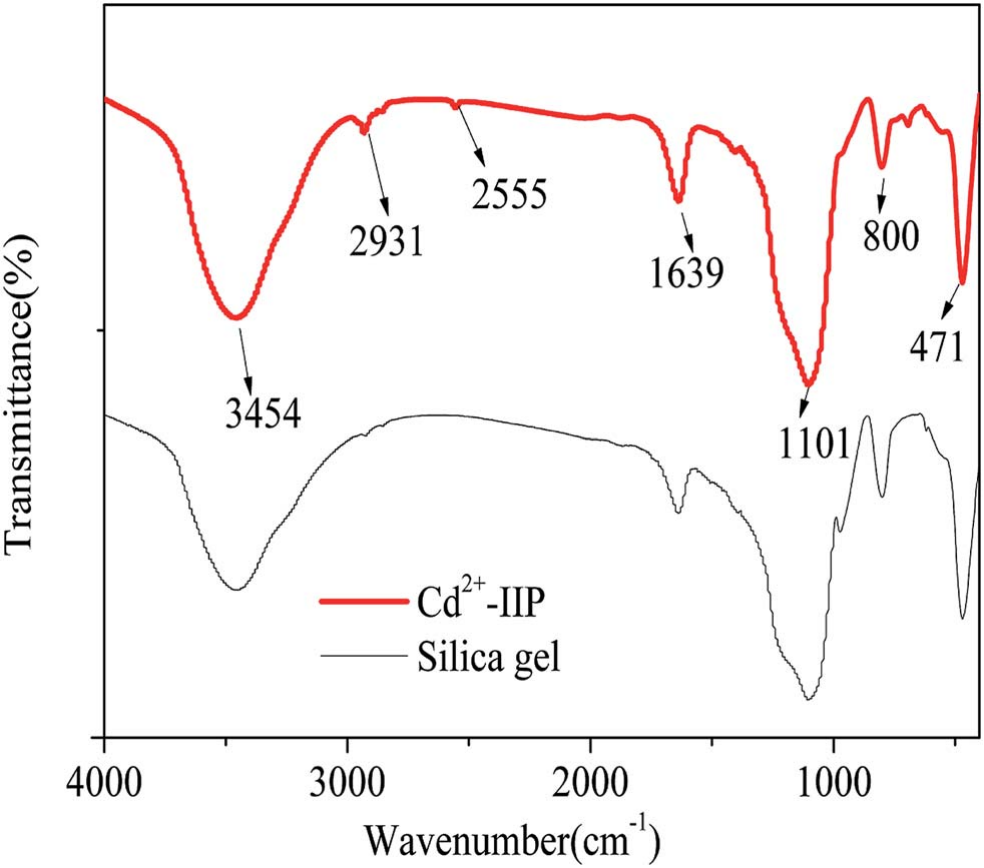
FT-IR spectra of Cd^2+^-IIP adsorbent and silica gel.

FT-IR spectra of silica gel and Cd^2+^-IIP are shown in Fig. 4. The peaks at 3454 and 1639 cm^−1^ correspond to the vibrations of ≡Si–OH and –OH in physisorbed water, respectively.^31^ The peak at 471 cm^−1^ was assigned to ≡Si–O–Si≡ stretching vibration,^31^ thus confirming the occurrence of silica matrices in the raw material. Changes in the FT-IR spectra were observed in Cd^2+^-IIP at the wavenumbers of 2931 and 2555 cm^−1^ attributed to the stretching vibration of –CH_2_ and –SH groups, respectively: band characteristic of –SH are known to fit into 2600–2450 cm^−1^.^32^ A broad absorption peak appeared at 1101 cm^−1^ corresponds to siloxane vibration of (SiO)_*n*_.^33^ The observed peaks consistently revealed MPTS successfully grafted onto the surface of silica gel in the imprinting processes.

#### Surface area and pore size analysis

**Fig. 5:**
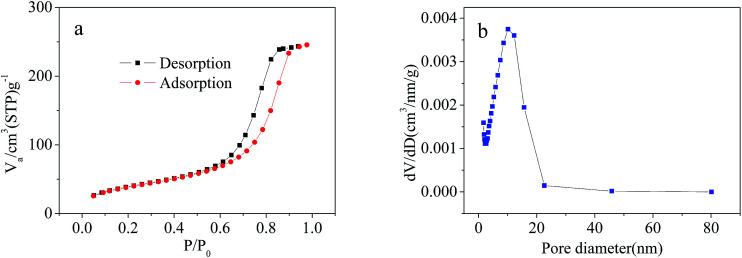
N_2_ adsorption–desorption isotherms of Cd^2+^-IIP at 77.3 K (a) and pore-size distribution curve of Cd^2+^-IIP (b).

As shown in Fig. 5(a), the N_2_ adsorption–desorption isotherm of Cd^2+^-IIP can be categorized as type IV, meaning that Cd^2+^-IIP was a mesoporous structure adsorbent. The surface area of Cd^2+^-IIP calculated from Brunauer–Emmett–Teller (BET) was 197.6 m^2^ g^−1^ which was due to the specific recognition cavities for Cd^2+^ ions created on the sorbent surface. According to Fig. 5(b), the average pore diameter of Cd^2+^-IIP was 10.2 nm which could be obtained from Barrett–Joyner–Halenda (BJH) model. The pore size distributions of Cd^2+^-IIP was also mainly in the scope of mesopores with 2–50 nm.

#### XPS study

**Fig. 6:**
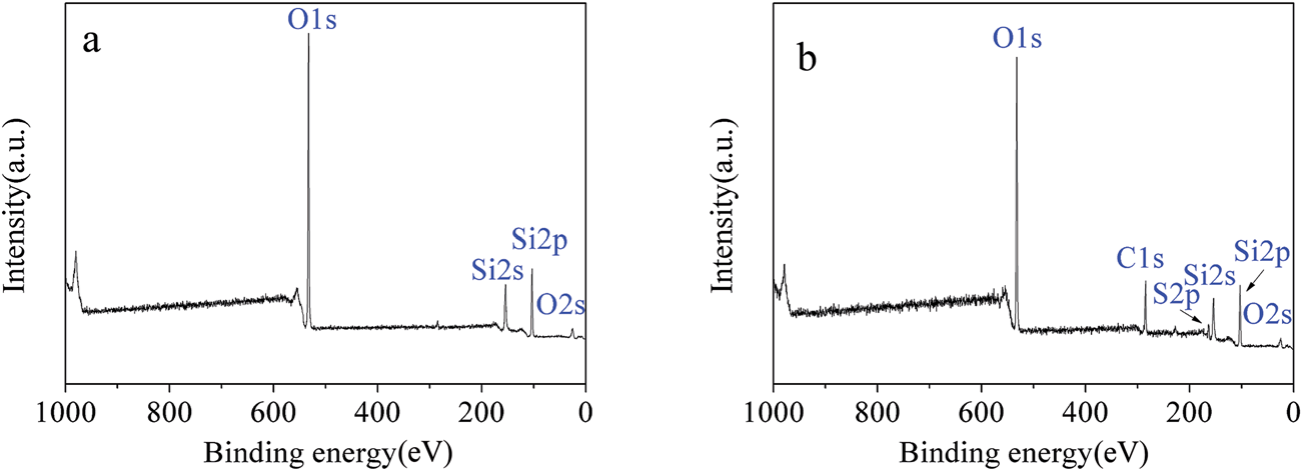
XPS survey spectra of silical gel and Cd^2+^-IIP.

The full scan XPS spectrums showed the presence of Si at the binding energies of 156.85 eV and 105.7 eV for silica gel (Fig. 6(a)), and 164.6 eV and 105.7 eV (Fig. 6(b)) for Cd^2+^-IIP, respectively. The element of O could also be seen at the binding energy of 26.4 eV for silica gel and 29.1 eV for Cd^2+^-IIP, respectively. After modification, the elements of C and S were appeared in the XPS spectra of Cd^2+^-IIP, indicating the successful introduction MPTS onto silica gel. The content of S obtained from XPS study was 5.92% which was close to the results of EDS study. Herein, the theoretical content of thiol group was 1.85 mmol g^−1^, and the amount of MPTS anchored onto silica surface was about 36.3%.

### Adsorption of Cd^2+^ at Cd^2+^-IIP: effect of pH and competing heavy metals

#### Adsorption of heavy metals from single-metal solutions

**Fig. 7:**
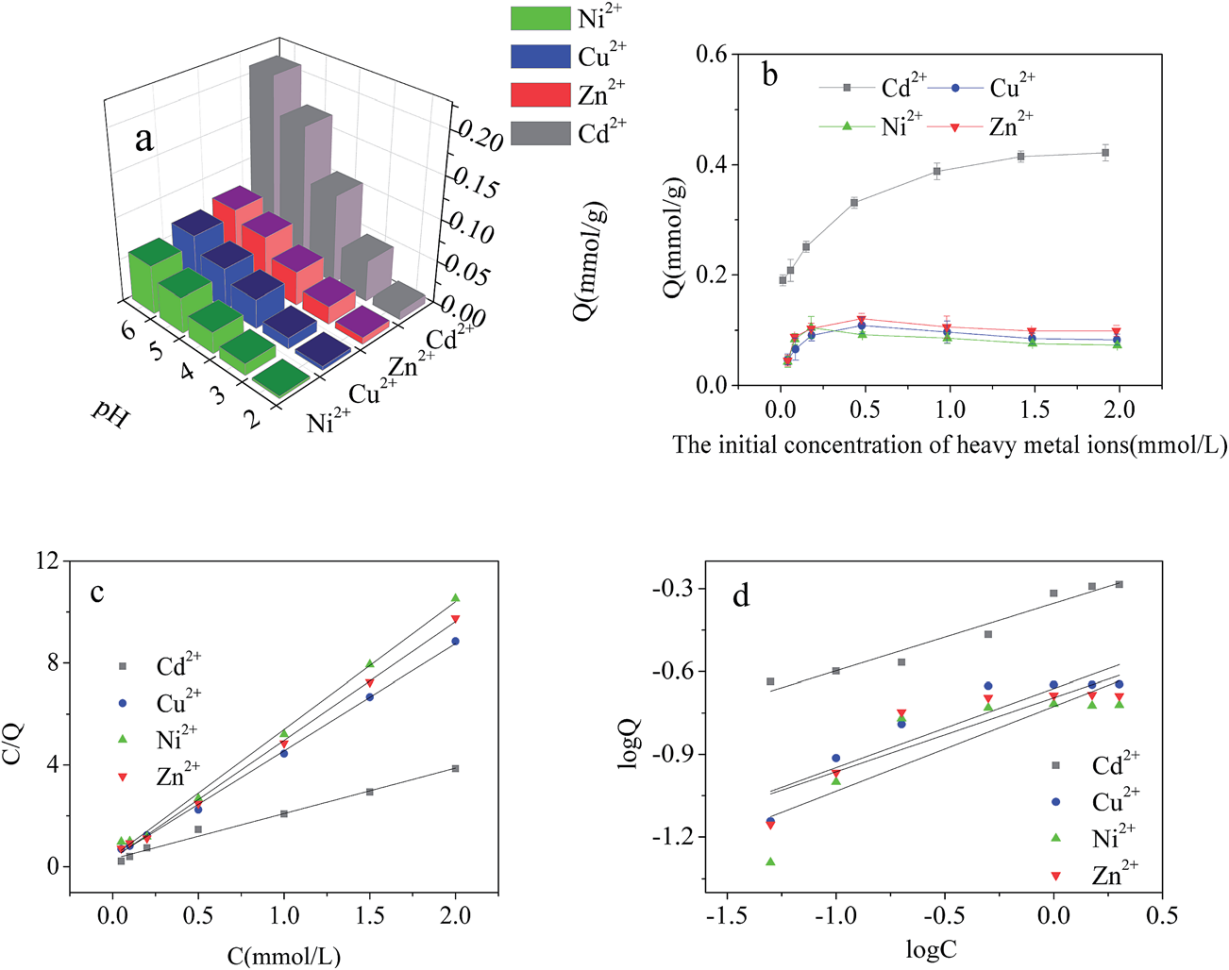
Effect of pH on the adsorption of Cd^2+^, Cu^2+^, Ni^2+^ and Zn^2+^ on Cd^2+^-IIP in single-metal solutions (a): *C*_0_ = 10 mg L^−1^, temperature = 25 °C, *t* = 4 h, the dosage of adsorbent = 0.02 g; dependence of Cd^2+^, Cu^2+^, Ni^2+^ and Zn^2+^ adsorption on the Cd^2+^-IIP adsorbent in single-metal solutions on the metals initial aqueous concentrations (b): pH = 5, *C*_0_ = 10–200 mg L^−1^, the dosage of adsorbent = 0.02 g, temperature = 25 °C, time = 4 h; the equilibrium isotherms: (c) the Langmuir model (d) the Freundlich model.

pH as a factor controlling the surface charge of the adsorbent and the ionization degree of the heavy metal ions is the most important factor of adsorption.^34,35^ Its study in the present research was realized within the pH range from 2.0 to 6.0 to avoid precipitation of heavy metal ions at higher values. The effect of pH on the adsorption capacity of Cd^2+^-IIP with regards to Cd^2+^, Cu^2+^, Ni^2+^ and Zn^2+^ in single-ion solutions is given in Fig. 7(a). It can be seen that the adsorption capacity increased with the pH: at low pH the protonation of adsorption sites and the net positive charge of the surface hamper heavy metal ions from approaching the surface of Cd^2+^-IIP. The affinity of Cd^2+^-IIP towards the ions lined up in the order Cd^2+^ > Cu^2+^ > Zn^2+^ > Ni^2+^ with the one to Cd^2+^ substantially higher than the others attributed to the pre-designed matching of Cd^2+^-IIP for Cd^2+^. The cationic radii of Cd^2+^, Zn^2+^, Cu^2+^ and Ni^2+^ was 0.97, 0.74, 0.73 and 0.69 Å,^36^ respectively, which was almost unanimous with the above order. Zn and Cu have radii pretty close to each other thus making the difference for the single- and binary-metal solutions minimal with the potential error of measurement.

The effect of initial concentration of heavy metal ions on the adsorption capacity of Cd^2+^-IIP in single-metal solutions are given in Fig. 7(b). The selective character of the ion-imprinted Cd^2+^-IIP adsorbent in respect to Cd^2+^ was thus confirmed. For the purpose of fully understanding the adsorption behavior of Cd^2+^, Cu^2+^, Ni^2+^ and Zn^2+^ onto Cd^2+^-IIP in single system, Langmuir and Freundlich isotherms was used to fit the experimental data and the results were shown in Table 1 and Fig. 7(c, d). The high correlation coefficients (*R*^2^ ≥ 0.981) indicated that the adsorption of Cd^2+^, Cu^2+^, Ni^2+^ and Zn^2+^ onto Cd^2+^-IIP in compliance with Langmuir isotherm. The adsorption capacities of Cd^2+^-IIP towards Cd^2+^, Zn^2+^, Cu^2+^ and Ni^2+^ in Langmuir model were also close to the experimentally obtained values. According to the assumptions of Langmuir isotherm model,^29^ monolayer adsorption of Cd^2+^, Cu^2+^, Ni^2+^ and Zn^2+^ happened on the surface of Cd^2+^-IIP, and it was mainly chemical adsorption.

**Table 1:** Parameters of Langmuir and Freundlich isotherms for adsorption of Cd^2+^, Cu^2+^, Ni^2+^ and Zn^2+^ on Cd^2+^-IIP in single system

Adsorbate	*Q*_exp_/(mmol g^−1^)	Langmuir isotherm			Freundlich isotherm		
		*Q*_max_/(mmol g^−1^)	*K*_L_/(L mmol^−1^)	*R* ^2^	*K*_F_	*n*	*R* ^2^
Cd^2+^	0.521	0.560	5.911	0.981	0.444	3.836	0.942
Zn^2+^	0.126	0.215	14.899	0.998	0.202	3.724	0.785
Cu^2+^	0.137	0.238	11.941	0.999	0.218	3.494	0.847
Ni^2+^	0.101	0.199	12.791	0.997	0.188	3.258	0.739

#### Adsorption of heavy metals from binary-metal solutions

**Fig. 8:**
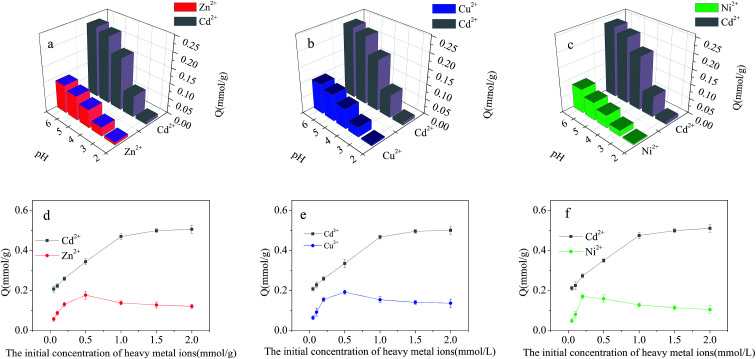
Effect of pH on the adsorption of Cd^2+^, Cu^2+^, Ni^2+^ and Zn^2+^ on Cd^2+^-IIP in binary-metal solutions: (a) Cd^2+^/Zn^2+^, (b) Cd^2+^/Cu^2+^, and (c) Cd^2+^/Ni^2+^(*C*_0_ = 10 mg L^−1^, temperature = 25 °C, *t* = 4 h, the dosage of adsorbent = 0.02 g); dependence of Cd^2+^, Cu^2+^, Ni^2+^ and Zn^2+^ adsorption on the Cd^2+^-IIP adsorbent in binary-metal solutions on the metals initial aqueous concentrations: (d) Cd^2+^/Zn^2+^, (e) Cd^2+^/Cu^2+^, (f) Cd^2+^/Ni^2+^ (pH = 5, *C*_0_ = 10–200 mg L^−1^, the dosage of adsorbent = 0.02 g, temperature = 25 °C, time = 4 h).

The impact of pH on the adsorption in binary-metal solutions is shown in Fig. 8(a–c). Being negligible at pH 2.0, the difference in adsorption capacities of Cd^2+^-IIP towards Cd^2+^ and the competing ions showed a substantial increase at higher pH. Compared with the single-metal solutions, the adsorption capacity in binary solutions decreased in respect to all metals, having, however, the adsorption capacity towards Cd^2+^ remained the highest as for the pre-designed imprinted polymer to fit the adsorption sites to the target ions.^37^ Based on the prerequisite, the other heavy metals presumably compete for the nonspecific hydroxyl group sites of Cd^2+^-IIP in ion exchange and the electrostatic interaction. The order of the Cd^2+^-IIP affinity towards the metal ions in binary-metal solutions follows the one observed in the single-metal ones. The selectivity coefficients of Cd^2+^-IIP for Cd^2+^ in the binary solutions are given in Table 2 at pH 5.0. It can be seen that the selectivity in the ionic pairs followed the descending order Ni^2+^ > Zn^2+^ > Cu^2+^. It should be noticed that although Cu^2+^, Ni^2+^ and Zn^2+^ ions have their charge and size close to those of Cd^2+^ at high affinity to the sulfhydryl ligand used in the Cd^2+^-IIP adsorbent, the latter still exhibits high selectivity towards Cd^2+^ for the specific recognition cavities designed in the template synthesis.

**Table 2:** The selectivity parameters of Cd^2+^-IIP for Cd^2+^ in binary system

Metals	*K*_d_(Cd^2+^)	*K*_d_(X^2+^)	*k*
Cd^2+^/Cu^2+^	4012	1073	3.74
Cd^2+^/Ni^2+^	4025	702	5.73
Cd^2+^/Zn^2+^	3983	959	4.15

The dependence of metallic ions adsorption at the Cd^2+^-IIP surface on their initial aqueous concentrations in binary-metal solutions (Cd^2+^/Cu^2+^, Cd^2+^/Ni^2+^, Cd^2+^/Zn^2+^) is given in Fig. 8(d, e and f), respectively. Similar to the single-metal solution, the adsorption of Cd^2+^ increased with increasing initial concentration of Cd^2+^. Unlike Cd^2+^, the adsorption Cu^2+^, Ni^2+^ and Zn^2+^ reached a maximum with further decrease and stabilization at a lower level with increasing aqueous concentration. At a relatively low concentration of contaminants, there was no competition between the metals, all the pollutants completely adsorbed on the surface of Cd^2+^-IIP, showing the adsorption equal to the one observed with the single-metal solutions. However, with the increasing initial concentration of metallic ions the competition was observed: Cu^2+^, Ni^2+^ and Zn^2+^ occupying the adsorption sites were substituted by Cd^2+^, making the surface concentrations of the ions competing Cd^2+^ decreased. This observation confirms the high selectivity of Cd^2+^-IIP towards Cd^2+^ originated from the template synthesis designing the specific adsorption sites for Cd^2+^ ions.

#### Adsorption of heavy metals from ternary- and quaternary-metal solutions

**Fig. 9:**
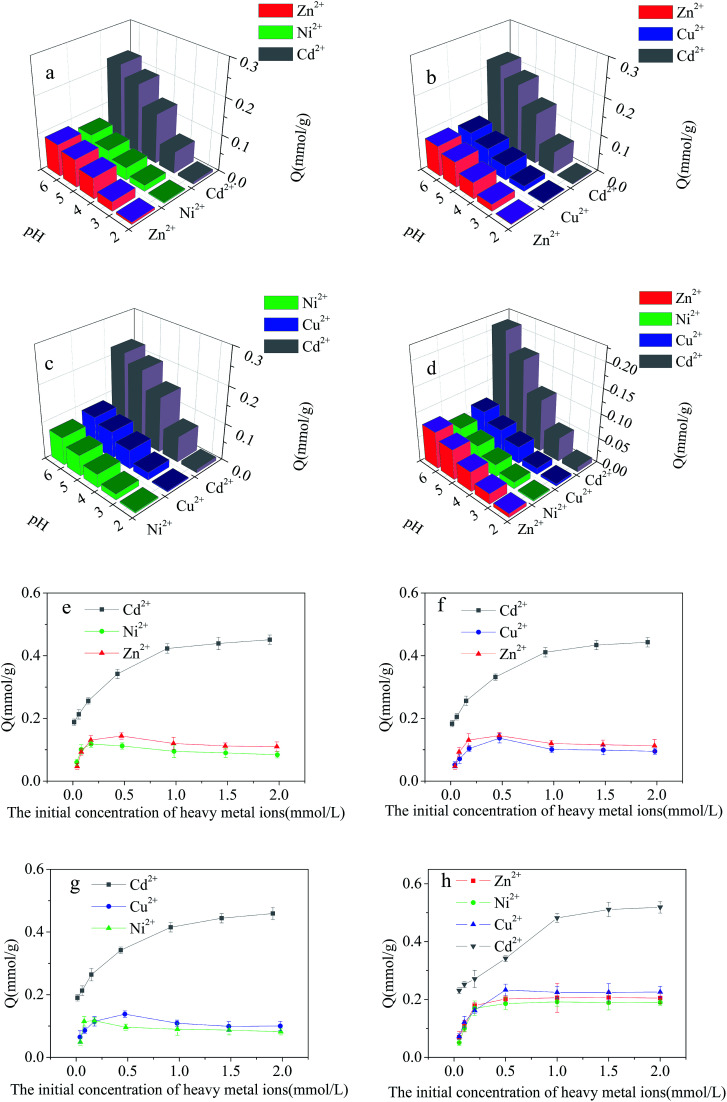
Effect of pH on the adsorption of Cd^2+^, Cu^2+^, Ni^2+^ and Zn^2+^ on Cd^2+^-IIP in ternary- and quaternary-metal solutions: (a) Cd^2+^/Zn^2+^/Ni^2+^, (b) Cd^2+^/Zn^2+^/Cu^2+^, (c) Cd^2+^/Cu^2+^/Ni^2+^ and (d) Cd^2+^/Zn^2+^/Cu^2+^/Ni^2+^(*C*_0_ = 10 mg L^−1^, temperature = 25 °C, *t* = 4 h, the dosage of adsorbent = 0.02 g); dependence of Cd^2+^, Cu^2+^, Ni^2+^ and Zn^2+^ adsorption on the Cd^2+^-IIP adsorbent in ternary- and quaternary-metal solutions on the metals initial aqueous concentrations: (e) Cd^2+^/Zn^2+^/Ni^2+^, (f) Cd^2+^/Zn^2+^/Cu^2+^, (g) Cd^2+^/Cu^2+^/Ni^2+^ and (h) Cd^2+^/Zn^2+^/Cu^2+^/Ni^2+^(pH = 5, *C*_0_ = 10–200 mg L^−1^, the dosage of adsorbent = 0.02 g, temperature = 25 °C, time = 4 h).

The effect of pH on the adsorption results from the ternary- (Cd^2+^/Cu^2+^/Ni^2+^, Cd^2+^/Cu^2+^/Zn^2+^ and Cd^2+^/Zn^2+^/Ni^2+^) and quaternary-metal (Cd^2+^/Cu^2+^/Ni^2+^/Zn^2+^) solutions are given in Fig. 9(a–d). Compared with the results obtained with the binary-metal solutions, the adsorption capacity of Cd^2+^-IIP adsorbent in respect to the heavy metal ions decreased to a various extent in the ternary- and quaternary-metal solutions. The affinity order also somewhat changed: the adsorption capacity descent observed in the row Cd^2+^ > Zn^2+^ > Cu^2+^ > Ni^2+^ being different from the observed for the binary-metal solutions.

The effect of initial concentration of heavy metal ions on the results of the competitive adsorption from ternary- and quaternary-metal solutions onto Cd^2+^-IIP adsorbent are given in Fig. 9(e–h). The amount of Cd^2+^ adsorbed by Cd^2+^-IIP in multi-metal solutions exceeded the ones of other heavy metal ions at equal starting concentration. As for the other heavy metals, the adsorption isotherms of these lined up in adsorbed quantities in the descending order of Zn^2+^ > Cu^2+^ > Ni^2+^ consistent with cationic radii,^36^ respectively. The ion radius is thus apparently playing an important role in the selective adsorption performance of ion-imprinted Cd^2+^-IIP adsorbent, making the observation potentially useful in designing of adsorbent materials with the specific properties.

### Effect of organic acids on adsorption of Cd^2+^

#### Adsorption of organic acids on Cd^2+^-IIP

**Fig. 10:**
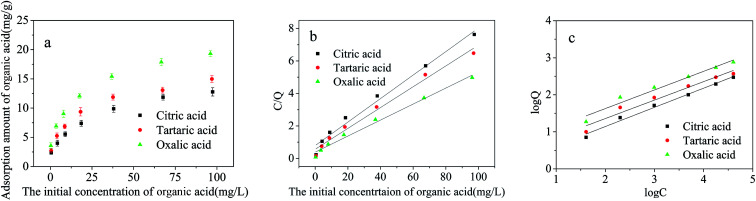
(a) Dependence of citric, tartaric and oxalic acid adsorption on the Cd^2+^-IIP adsorbent on their initial aqueous concentrations (pH = 7, *C*_0_ = 1–100 mg L^−1^, the dosage of adsorbent = 0.02 g, temperature = 25 °C, *t* = 4 h); the equilibrium isotherms: (b) the Langmuir model (c) the Freundlich model.

When the concentration of low molecule weight organic acids was in the range of 0–100 mg L^−1^, the adsorption performance of citric, tartaric and oxalic acids on the Cd^2+^-IIP adsorbent from single-acid solutions in pH = 7 is illustrated in Fig. 10(a). The affinity of acids to Cd^2+^-IIP followed the descending order oxalic > tartaric > citric acid, consistent with their molecular mass growth: the smaller the molecular weight of the acid the more of it adsorbs on the Cd^2+^-IIP surface by hydrogen bond, van der Waals' force and/or electrostatic interaction. Hydrogen bond is one of the most important interaction force in the adsorption process of silica gel which has hydroxyl groups and silanol groups.^38^ Cd^2+^-IIP contain –OH and –SH group, while the organic acid contain –COOH and –OH group, so hydrogen bond may exist in the adsorption process of Cd^2+^-IIP towards organic acids. Electrostatic interaction also plays an important role in the acids adsorption by Cd^2+^-IIP. Generally, the adsorbed amounts of the acids were relatively low which might be due to the negative charge of dissociated acid anions^39^ repelled by thiol-functionalized group so it might have a relatively strong repulsion forces with Cd^2+^-IIP. According to previous report, oxalate, tartrate and citrate ions can form surface chelates with 5- or 6-memberated ring structures, which are far more stable than mono-dentate complexes.^39^ So oxalate, tartrate and citrate ions may chelate the surface of Cd^2+^-IIP with 5- or 6-memberated ring structures. The chelation may also be therefore responsible for the acids adsorption.

The parameters of Langmuir and Freundlich isotherms of small molecular organic acids are given in Table 3 and Fig. 10(b, c). Since the correlation coefficients (*R*^2^) of Freundlich model exceeding 0.99 surpass those of the Langmuir one, the adsorption seems to follow the multilayer pattern^40^ with consistently increasing surface acid concentration with the increased aqueous concentration of adsorbate.

**Table 3:** Parameters of Langmuir and Freundlich isotherms for adsorption of citric acid, tartaric acid and oxalic acid onto Cd^2+^-IIP

Adsorbate	Langmuir isotherm			Freundlich isotherm		
	*Q*_max_/(mg g^−1^)	*K*_L_/(L mg^−1^)	*R* ^2^	*K*_F_	*n*	*R* ^2^
Citric acid	14.104	0.076	0.984	2.296	2.612	0.996
Tartaric acid	15.949	0.090	0.988	2.858	2.693	0.992
Oxalic acid	20.921	0.091	0.989	3.749	2.691	0.994

#### Effect of organic acids on Cd^2+^ adsorption isotherms

**Fig. 11:**
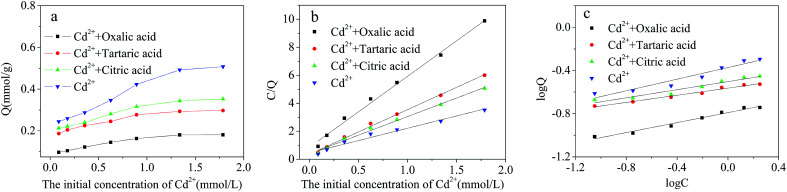
(a) Dependence of Cd^2+^ adsorption on the Cd^2+^-IIP adsorbent on its initial aqueous concentration in presence of citric, tartaric and oxalic acids in single-acid solutions (pH = 7, *C*_0_ = 10–100 mg L^−1^, the dosage of adsorbent = 0.02 g, temperature = 25 °C, *t* = 4 h); the equilibrium isotherms: (b) the Langmuir model (c) the Freundlich model.

The effect of citric, tartaric and oxalic acid admixtures in binary solutions on the adsorption capacity of Cd^2+^ is shown in Fig. 11(a). The initial concentration of acids was kept at 50 mg L^−1^ and the initial concentration of Cd^2+^ ranged from 0 to 200 mg L^−1^. Compared with the adsorption of Cd^2+^ from the single-metal solution, the adsorption capacity of Cd^2+^-IIP adsorbent towards Cd^2+^ in presence of citric, tartaric and oxalic acids decreased for 32.9%, 43.9% and 64.7%, respectively. Strong impact of acids is seen in the ability of these to form stable complexes with metallic ions obstructing adsorption of free metal cations at the adsorbent surface. The difference between acids is explained due to the molecular size, the number of carboxyl moieties and electric charge characteristics of the organic acids, determining stability of their complexes with Cd^2+^. From the parameters of isotherm models (Table 4 and Fig. 11(b, c)). It can be seen that the Langmuir model fitting to the experimental Cd^2+^ adsorption data characterizing the surface of the adsorbent as uniform and energetically homogeneous^41^ providing monolayer adsorption of Cd^2+^. The adsorption capacities of Cd^2+^-IIP towards Cd^2+^ in presence of the acids of the Langmuir model were also close to the experimentally obtained values.

**Table 4:** Parameters of Langmuir and Freundlich isotherms for adsorption of Cd^2+^ onto Cd^2+^-IIP in the presence of citric acid, tartaric acid and oxalic acid

Coexisting organic acids	Langmuir isotherm				Freundlich isotherm		
	*Q*_exp_/(mmol g^−1^)	*Q*_max_/(mmol g^−1^)	*K*_*L*_/(L mmol^−1^)	*R* ^2^	*K*_F_	*n*	*R* ^2^
Control group (Cd^2+^)	0.508	0.570	3.837	0.976	0.139	3.719	0.936
Citric acid	0.353	0.377	6.780	0.993	0.070	5.336	0.949
Tartaric acid	0.297	0.314	8.953	0.997	0.051	6.045	0.982
Oxalic acid	0.181	0.197	5.989	0.994	0.013	4.325	0.977

#### Effect of the initial concentration of organic acids on the adsorption of Cd^2+^

**Fig. 12:**
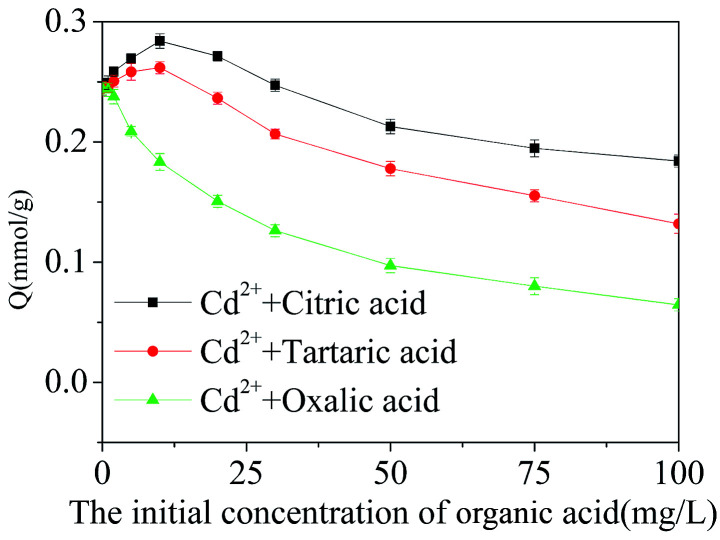
Effect of initial concentrations of organic acids on the adsorption of Cd^2+^ on Cd^2+^-IIP adsorbent (pH = 7, *C*_0_(Cd^2+^) = 10 mg L^−1^, the dosage of adsorbent = 0.02 g, temperature = 25 °C, *t* = 4 h).

The adsorption of Cd^2+^ decreasing in presence of organic acids (Fig. 11) requires closer insight to characterize the impact of acid admixtures to the adsorbent performance. Fig. 12 presents the dependence of Cd^2+^ adsorption from the solutions containing 10 mg L^−1^ of the metal cation on the content of organic acids (10–100 mg L^−1^). The adsorption of Cd^2+^ increased with the increasing initial concentration of citric and tartaric acids in single-acid solutions from 0 to 10 mg L^−1^, and then decreased with the further increasing acid concentration. The adsorption of Cd^2+^ was, however, only reduced in the presence of oxalic acid, the increase in adsorption was not observed. The inhibitory effect of acids strengthened with the increased acid concentration in respect of all acids. Moreover, from the achieved results, it is possible to affirm that IIP is prone to interference by matter organic and can not be used for Cd^2+^ removal from real samples when the initial concentration of organic acid was higher than 10 mg L^−1^.

The adsorption ability of Cd^2+^-IIP mainly depends on the active adsorption sites, electric charge of adsorbent surface and the properties of adsorbate. Organic acids contain carboxyl groups, which have the ability to coordinate with Cd^2+^-ions exhibiting their cumulative complexing stability constants in the descending order tartaric > citric > oxalic acid.^42,43^ The role of citric acid and tartaric acid in promoting the adsorption of Cd^2+^ may be due to (i) formation of water-soluble Cd^2+^-complexes of organic acids having the electric charge contrary to the one of adsorbent, and, probably, forming a dipole with its charge polarization,^44^ and/or (ii) formation of outer sphere Cd^2+^–acid complexes at the Cd^2+^-IIP adsorption sites: the multi-layer adsorption of acids described by the Freundlich model indirectly supports this hypothesis (Fig. 10 and Table 4). The Cd^2+^ adsorption inhibited with acids may be explained by the discussed above formation of water-soluble Cd^2+^–acid complexes.^41^

### Regeneration

**Fig. 13:**
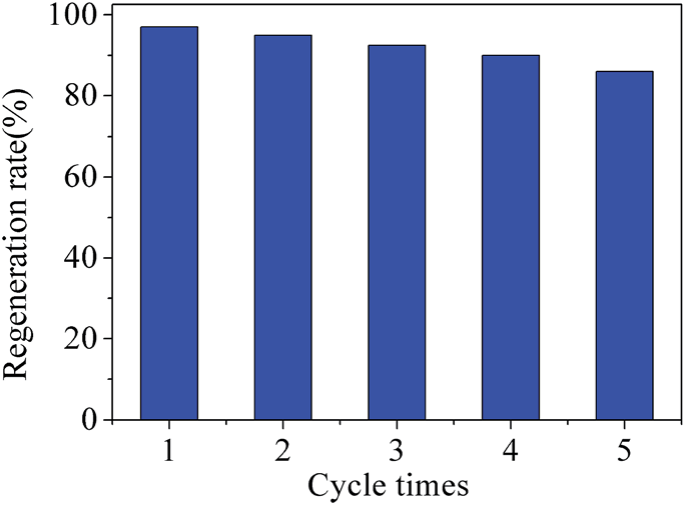
Recycling of Cd^2+^-IIP in the removal of Cd^2+^ from aqueous solutions (*C*_0_(Cd^2+^) = 0.1 mmol L^−1^, the dosage of adsorbent = 0.02 g, temperature = 25 °C, *t* = 4 h).

The reuse ability of one adsorbent is a very important parameter for its in practical application.^45^ Therefore, the regeneration performance of Cd^2+^-IIP with thiol-functional groups was investigated. 0.02 g Cd^2+^ ion-imprinted adsorbent was immersed into 100 mL Cd^2+^ solution with the concentration of 0.1 mmol L^−1^ and stirred for 4 h at 25 °C. After adsorption, the used Cd^2+^-IIP was dried by filtration and isolation and then added to the conical flask with 1 mol L^−1^ HCl solution. Fig. 13 gave some details for regeneration experiment results. After reused for 5 times, Cd^2+^-IIP still showed good regeneration rate which was greater than 85%, indicating that Cd^2+^-IIP was a potential adsorbent for practical application.

## Conclusions

The Cd^2+^ ion-imprinted adsorbent Cd^2+^-IIP with thiol-functional groups was successfully synthesized applying the surface ion imprinting technique combined with ultrasonic heating and hydrothermal method using silica gel as substrate. The Cd^2+^-IIP adsorbed the target Cd^2+^-ion from aqueous solutions with high degree of selectivity. The latter was achieved on account of Cd^2+^ interaction with specific proper-size recognition cavities in Cd^2+^-IIP adsorbent originated from the synthetic procedure: the template synthesis left Cd^2+^-ion proper size voids in the carrier material. The selectivity was demonstrated in respect to mixtures with Zn^2+^, Cu^2+^ and Ni^2+^ ions of the size and charge close to the target Cd^2+^. The description of metal ions adsorption at the Cd^2+^-IIP adsorbent is satisfactorily described by the Langmuir model. The presence of tartaric, citric and oxalic acids as admixtures in Cd^2+^ aqueous solutions noticeably reduced the cation adsorption in wide range of concentrations with the minor exception of low contents of citric and tartaric acids slightly improving adsorption. The impairment of adsorption may be explained by formation of poorly adsorbable water-soluble complexes switching off the template-designed Cd^2+^-size cavities from adsorption. Preliminary decomposing of metal–acid complex compounds with, *e.g.* oxidation, liberating free metallic cations prior to adsorption may appear necessary.

## Conflicts of interest

There are no conflicts to declare.

## Supplementary Material
